# Abortion

**Published:** 1892-11

**Authors:** R. G. Webster


### ABORTION.*

By R. G. Webster, V.M.D.

Synonyms: Slipping, dropping, slinking. When pregnancy is interrupted by the expulsion of the ovum or fœtus before it has attained sufficient development to live external to its parent, abortion is said to occur, but when the fœtus is expelled before the ordinary period, yet with the organs sufficiently developed to live for at least a time, premature birth is said to have taken place; in our practice there is no well defined limit between abortion and premature birth. Abortion may occur at any time during pregnancy, up to within a month of the normal time. It is more often seen during the first half of pregnancy, the ovum often escaping without being noticed by the owner, or affecting the health of the animal. In the later stage, or last few months, after the fœtus has developed to some size, we often have serious trouble, not only in delivering the calf, but also incurring a loss to the owner as a milch cow, she seldom doing so well that season, and is very likely to go through the same performance the next time she gets pregnant.

Causes: These are many and are divided into two classes; (1) sporadic, or accidental; (2) enzootic, or epizootic. When cases occur here and there over a wide extent of country without any relationship as to the cause, they are termed sporadic, or accidental. The causes of these of these are numerous, and may be classified as external and internal causes. External causes: atmospherical changes, irregular seasons, sudden changes from hot to cold, or vice versa. Many observers have noticed that a long continued cold or hot spell is less productive to this complaint than one wet, cold or frosty night in Autumn succeeding a hot day. A very frequent cause is in the food; where the food is of bad quality, not being harvested properly; may be due to too much rich food, causing an accumulation of fat in the uterus. I myself had a case only a few weeks ago due to this cause; the fatty matter had accumulated in lower portion of uterus, and had loosened the fœtal membranes from the cotyledons. Bad, filthy water, which our cattle are often compelled to drink, drainage water which has stood until it is stagnant. Blows upon the belly from other cattle, or attendants, jamming through narrow passage ways, being knocked about in transportation, excessive exercise,

* Paper presented at the September meeting of the Keystone Veterinary Medical Associa tion sudden fright, excitement from being chased by dogs, violent purges.

Internal causes : In some animals there appears to be a tendency to abort more than in others ; they do so without any apparent cause, and without any constitutional trouble being noticed. The most constant internal cause is from some disease of the animal, where she has had a high fever for some time, and where there is coughing, viz.: as in bronchitis, pneumonia, pleurisy, tuberculosis and other diseases where the pain is excessive. A tubercular condition of the uterus I believe is a very frequent cause, as it is often noticed in animals of this class.

Symptoms : The symptoms vary ; in some they are so slight as to passed unperceived by any one, while in others they are very serious ; this usually depends upon the time that abortion takes place, whether at first or second stage of gestation. When abortion takes place during the first few months, the cow may appear to the stableman as well as any of his herd up to within a short time of aborting ; ofttimes when this trouble happens at night there is no sign the evening before, she eating and milking as usual, and the owner is often at a loss when finding a calf behind his cows to know to which it belongs, as the mother will take no notice of it whatever, often trampling it under her feet, and showing no signs of distress. If the cow is noticed by an expert the day before dropping her calf, he will detect an enlargement of the vulva, with slight falling in of the flanks, and some uneasiness upon the part of the mother, at times a discharge from vulva of a thick ropy fluid. In what is termed the laborious or difficult abortion, which is caused by blows, kick, or hook from other animals, the symptoms are well marked, ofttimes causing a great amount of suffering, depending much upon whether the fœtus is alive or dead. In these cases the veterinarian is called in to see the animal with the history that she is due to calve in two or three months, has been feeding well until a few days before, when he noticed her looking dull and at times uneasy, lying down and getting up, looking around at her sides ; from her movements he thinks she has the colic ; upon examination by the veterinarian he will notice a peculiar expression about face and eyes, the pulse is quick, small and hard, respirations quickened, will give a strain with a groan ; at this time he may notice a whitish discharge which later turns to a yellow or reddish hue; by watching the abdomen he will see the fœtus, if alive, in violent motion. Soon after this we will have the symptoms of a normal parturition, viz.: the uterus begins to contract, the expiratory muscles acting in conjunction therewith. The extent of these labor pains depends much upon the strength of the animal, and suddenness of the attack. The first result of the straining is the emptying of the rectum and bladder, next dilation of os uteri and protrusion of membranes into the vagina, then through vulva externally; as the water bag ruptures the pains are excessive and powerful, soon expelling fœtus, sometimes with all the membranes, but oftener they have to be removed by the hand with difficulty, where the animal is poor and weak external force is often necessary.

Epizootic, or infectious abortion: Epizootic, or infectious, differs much from the sporadic form in affecting whole herds of pregnant animals in one locality, and will occur year after year without any abatement, in spite of anything that can be done, and even without finding any cause for the outbreak, thus making it a great scourge to cattle raisers and farmers.

The causes that have been advanced are very conflicting. As long ago as the last century contagion or infection was believed to be the principal cause of this trouble In my own practice I have a client who had a fine herd of grade cattle and knew nothing of this disease, never having anything of the kind in his stable. He went to a drove yard and bought a fine looking cow, which after being on his farm a few weeks aborted; within a few months all of his pregnant animals aborted, and have been doing so ever since. In experiments performed by Franck, of the Munich Veterinary School, it has been established by microscopical investigation that on the lining membrane of the vagina and that of the vulva there is constantly found a minute fungus mixed with the mucus, in every respect similar to the leptothrix buccalis, being in fact a kind of bacterium. Toward the period of parturition these bodies become abundant and appear to concur in the decomposition of the fœtal membranes and their expulsion. Franck has shown that by smearing the vaginal canal of a pregnant animal to a certain depth with this matter from the expelled membranes of one that has been diseased, abortion can be induced.

Symptoms: It is rarely that this form of abortion takes place before the fourth month of gestation, is mostly in fifth and sixth, but may be later. This form of abortion is mostly without any effort or excitement upon the part of the mother, the fœtus mostly still born. Diagnosis is sometimes easy, but often very difficult, as we are often asked by owner if such and such a cow is likely to abort, which is very hard, if not impossible to answer, as she may show no outward signs, but still may abort in a few days; this puts the veterinarian in a position to be condemned, for if he had made his diagnosis correctly at this time it might have been prevented. Another question asked is, has such a cow aborted? In the absence of fœtus or fœtal membranes it is also hard to answer correctly.

Treatment is preventative and remedial. Preventative is due to knowledge of the cause, if it can be found. First and most important is cleanliness. The shed or stable should be well ventilated and drained, allowing the air to pass through at least two hours every day; should be whitewashed and disinfected after an animal has aborted, so as to kill the germs and smell. The animal herself should be removed from the rest of the herd for three or four weeks, the stableman after tending to her should be careful and not go among the other cattle without going out in the fresh air for a while. If a cow shows any sign of aborting she should at once be removed by herself. Abortion can sometimes be prevented, if seen early enough, by giving narcotics and keeping perfectly quiet for a few days, giving light, sloppy food. If when we have arrived and found the water bag bursted, the only thing that can be done is to give the animal ample time to expel her offspring, which if we find she cannot do, help must be given. The afterbirth should be removed at once, as it is easier gotten away at this time and with less harm to the animal.

